# Isolated Crohn's Colitis: Is Localization Crucial? Characteristics of Pediatric Patients From the CEDATA–GPGE Registry

**DOI:** 10.3389/fped.2022.875938

**Published:** 2022-05-31

**Authors:** Lotta Elonen, Lena Wölfle, Jan de Laffolie, Carsten Posovszky, the CEDATA–GPGE-Study-Group, Tobias Schwerdt

**Affiliations:** ^1^Department of Pediatric and Adolescent Medicine, University Medical Centre Ulm, Ulm, Germany; ^2^Department of General Pediatrics and Neonatology, Justus-Liebig-University, Abteilung für allgemeine Pädiatrie und Neonatologie, Universitätsklinikum Giessen und Marburg, Giessen, Germany; ^3^University Children's Hospital - Zürich, Zürich, Switzerland

**Keywords:** IBD, pediatric, Crohn's disease, ulcerative colitis, Crohn's colitis, isolated colonic Crohn's

## Abstract

**Introduction:**

Pediatric patients with inflammatory bowel disease (IBD) are classified into Crohn's disease (CD), ulcerative colitis (UC), and unclassifiable (IBD-U). However, data provide evidence that ileal CD (L1) is distinct from colonic CD (L2). The aim of this study was to investigate the clinical features of isolated Crohn's colitis in a pediatric population.

**Material and Methods:**

Children who were prospectively included in the CEDATA–GPGE registry on diagnosis were compared according to the diagnosis of CD with L2 vs. L1 and ileocolonic (L3) involvement pattern as well as IBD-U and UC. The clinical significance of L2 was investigated with regard to extraintestinal manifestations, treatment, surgery, and disease activity.

**Results:**

Fifty-two patients with L2 CD at a median age of 13.4 years (±3.8 SD) were compared with 182 L1 (13.8 ± 2.9 SD), 782 with L3 (12.8 ± 3.3 SD), 653 with UC (12.7 ± 3.8 SD), and 111 patients with IBD-U (11.9 ± 4.7 SD). Bloody stools at diagnosis were more common in L2 (44%) than in L1 (19.7%) and L3 (28.8%), but not as common as in UC (66.5%) and IBD-U (61.3%). Fewer CD patients with L2 (10.2%) received exclusive enteral nutrition therapy (EEN) as induction than patients with L1 (34.3%) and L3 (33.3%). After induction therapy, 42.3% of patients with L2 received immunosuppressants and 21% biologicals during follow-up (L1 56.5/10.5%; L3 59/21%; CU 43.5/11.9%; IBD-U 26.1/12.6%). Extraintestinal manifestations were more frequent in L2 (23.1%) vs. L1 (18.7%), L3 (20.2%), CU (15.8%), and IBD-U (11.7%). The number of patients requiring surgery did not differ within the CD subgroups and was significantly lower in UC and IBD-U. Perianal fistula surgery was significantly more common in L2 (44%) than in L1 (4.8%) or L3 (21.7%). In addition, the frequency of surgery for perianal abscesses was also more frequent in L2 (55.6%) than in L1 (12.7%) or L3 (38.4%).

**Conclusions:**

The consideration of pediatric Crohn's colitis as a distinct disease seems necessary as it is characterized by extraintestinal manifestations (EIMs) with mainly joint involvement and perianal fistulas or abscesses requiring surgery and biologic therapy. Thus, colonic Crohn's disease may have an influence on the therapeutic stratification and should be addressed in further studies.

## Introduction

Comprehensive diagnostics must be performed to identify the disease phenotype according to the pediatric Paris classification (1). According to the guidelines, the diagnosis of inflammatory bowel disease (IBD) in children and adolescents includes history, clinical examination, and laboratory tests, as well as ileocolonoscopy and esophagogastroduodenoscopy (EGD) with stepwise biopsies (2, 3). In addition, imaging of the small bowel, preferably by magnetic resonance (MR) enteroclysis, is required. It is essential in Crohn's disease (CD) and unclassifiable IBD (IBD-U) and optional in ulcerative colitis (UC). According to the revised Porto criteria, patients with pediatric IBD (PIBD) are classified into CD, UC, and IBD-U (2). Crohn's disease is classified based on the pattern of involvement, as ileal with or without cecal involvement, defined as L1, isolated colon involvement called L2, or involvement of terminal ileum and colon called L3. Isolated colonic CD is diagnosed in only a smaller group of pediatric (13–24%) and adult patients (13–22%) (4–10). Because of the relative high incidence of atypical UC presentation in children with rectal sparing, cecal patch, skip lesions, and gastritis, the diagnostic differentiation in this group is especially important (11).

Data from the largest genotype–phenotype study in patients with IBD support the presence of a continuum of inflammatory bowel disease that is much better described by three groups (ileal Crohn's, Crohn's colitis, and ulcerative colitis) than the current binary classification into CD and UC (12). Disease localization is thus an essential aspect of a patient's disease, which is partly genetic and mainly responsible for the changes in disease behavior over time (12, 13). In recent years, there is increasing evidence that ileal and colonic CD differ in terms of epidemiology, genetics, macroscopic (such as creeping fat), and histologic findings, mucosal barrier, and infiltrating immune cells (13–16). The Crohn's subtypes also differ in the course of the disease with regard to disease progression after diagnosis (13). Furthermore, the groups differ in their response to therapeutic measures (7, 17, 18). Therefore, some authors proclaimed that an isolated Crohn's colitis should be considered “the third IBD” (13, 14, 19).

Data on the clinical significance of the CD phenotype in children and adolescents for disease progression and treatment are still scarce. The aim of this study was to investigate the clinical features of isolated Crohn's colitis in a large pediatric patient registry of the German-Speaking Society of Pediatric Gastroenterology and Nutrition (CEDATA–GPGE) with regard to (1) surgery, (2) long-term immunosuppressant therapy or biologic therapy, (3) extraintestinal manifestations, and (4) disease activity.

## Materials and Methods

### Study Cohort and Ethics

The CEDATA–GPGE is a prospective, multicenter German–Austrian registry for PIBD in German-speaking countries (20). The data of patients below the age of 18 years (at data entry) are prospectively collected since 2004. Here we evaluated 5,673 case records from 112 study centers until 8 December 2020. The patients' legal guardians gave their written consent to participate in the registry. The registry study was reviewed and approved by the ethic committee at the Faculty of Medicine of the Justus–Liebig University of Giessen and all participating institutions. No further ethical approval or consent was required for this study.

### Database Structure and Reporting Forms

The register was initiated and funded by the GPGE. From 2004 to 2010, the data was collected on forms and sent by post for the central data entry; thereafter the data entry was done *via* a secure online platform. Two reporting categories were recorded as follows: First, presentation and disease progression report. The first form was completed at admission to the registry and captured baseline data (e.g., age, gender, and family history of inflammatory bowel disease), diagnosis, examination findings, and initial symptoms (e.g., diarrhea, hematochezia) including extraintestinal manifestations as well as severity of disease and complications such as fistulae, stenosis, and growth retardation. The follow-up questionnaire was completed as often as possible during the doctor's visits but at least twice a year. It contained clinical information on the course of the disease, disease activity, examination findings (e.g., laboratory values, fecal calprotectin, height, and weight) and treatment measures.

### Diagnosis and Initial Treatment

The investigations to confirm the diagnosis were recorded in the registry, as well as the treatment for each of the first 3 months of induction therapy. The following exclusion criteria were formulated and applied for further analysis:

- Time interval of at least 3 months between diagnosis and initial report to the central office or at least 14 days between the initial report and the first documentation form for patients reported before 16 December, 2016, respectively.
- Missing or incomplete informed consent form.
- Missing diagnosis or diagnosis that cannot be clearly assigned in the course of treatment.

The total number of patients after applying the exclusion criteria was 2,122.

Furthermore, these patients were divided into subgroups according to diagnosis (CD, UC, and IBD-U). In the case of CD, a distinction was also made according to the pattern of disease (L1/L2/L3). The criteria of the Paris classification were applied:

- L1: infestation of the terminal ileum ± cecal infestation.
- L2: infestation of the ascending colon and/or transverse colon and/or descending colon
- L3: Infestation of the terminal ileum, additional infestation of the ascending colon, and/or transverse colon and/or descending colon.

The classification according to the pattern of involvement was made by analyzing the documented findings after imaging diagnostics. Due to insufficient or missing information on the localization pattern of disease, a total of 1,780 patients were finally included in the analysis. Follow-up data were collected at 6 and 12 months after diagnosis ± 3 months.

### Statistical Analysis

Statistical analysis was performed using Statistical Package for Social Science (SPSS) (version 26, IBM, Germany). Categorical data were reported as count and percentage. Continuous data were reported as mean and standard deviation or median, minimum or maximum, or range, according to data distribution. For the group comparison in parametric data for independent samples, Student's *t*-test and, in appropriate situations, ANOVA were used. A statistical evaluation of the nominal data was carried out using a Chi-square test. In this way, the risk ratio could be calculated in suitable cases. An explorative *p* < 0.05 was considered statistically significant.

## Results

### Patient Clinical Characteristics

The data from 1,780 patients in the CEDATA–GPGE registry were analyzed. Of these, 182 patients (10.2%) had ileal Crohn's (L1), 52 patients (2.9%) had Crohn's colitis (L2), 782 (43.9%) had ileocolitis Crohn's (L3), 653 (36.7%) had ulcerative colitis (UC), and 111 (6.2%) had IBD-U. In most patients with L2 CD, inflammation was described as skip lesions at diagnosis (23/38, 60.5%), followed by an inflammation restricted to the left colon (8/38, 21.1%), or involving more (3/38, 7.9%) or all parts of the colon (4/38, 10.5%). The 552 patients with UC for whom localization information was available were distributed among the subgroups as follows: 6.9% E1, limited to the rectum; 17.8% E2, left-sided colitis; 9.1% E3, extensive colitis; and 66.3% E4, pancolitis. The baseline characteristics are shown in Table 1. There was no significant difference in age at the time of diagnosis and gender between subgroups L2 and L1 or L3, but patients with UC or IBD-U were significantly younger (*p* = 0.001). Complications at diagnosis were similarly frequent in 13.5% with L2, 12.1% with L1 and 12.3% with L3, and less frequent in CU (2.0%, *p* < 0.0001) and IBD-U (1.8%, *p* = 0.0053). Bloody stools at diagnosis were significantly more common at 45.1% in L2 than in L1 (20.2%, *p* = 0.0003) and L3 (29.9%, *p* = 0.023), but not as common as in UC (69.7%, *p* = 0.0002) and IBD-U (62.4%, *p* = 0.04). Bowel movements were more frequent in L2 and L3 than in L1 and almost as frequent as with UC (Table 1). Liquid stools were more frequent in UC (36,7%) and IBD-U (40%) than in L2 (18,8%) at diagnosis.

**Table 1:** Basic characteristics of patients according to their disease.

	**L1**	**L2**	**L3**	**UC**	**IBD-U**	***p*-value**
	**(*N* = 182)**	**(*N* = 52)**	**(*N* = 782)**	**(*N* = 653)**	**(*N* = 111)**	**(ANOVA)**
**Age at diagnosis, in years**						
Median (±SD)	13.75 (2.9)	13.42 (3.8)	12.75 (3.3)	12.67 (3.8)	11.92 (4.7)	*p* = 0.014 L1-3, *p* = 0.001 ALL
Range	4.50–17.67	1.75–17.75	0.25–17.75	0.84–17.95	0.41–19.50	
**Observation period in months**						
Median (±SD)	18.5 (28.5)	30.0 (27.6)	24.0 (30.1)	24.0 (28.9)	11.0 (23.0)	*p* = 0.003 L1-3, *P* < 0.0001 ALL
**Sex at diagnosis**						
Male [*n* (%)]	101 (55.5%)	28 (53.8%)	461 (59.0%)	319 (48.9%)	68 (61.3%)	*p* = 0.557 L1-3, *p* = 0.002 ALL
**Complications with diagnosis**	22 (12.1%)	7 (13.5%)	96 (12.3%)	13 (2.0%)	2 (1.8%)	*p* = 0.98 L1-3, *P* < 0.0001 ALL
**Blood in the stool (** ***n*** **=)**	178 (98%)	51 (98%)	753 (96%)	623 (95%)	109 (98%)	
Yes	36 (20.2%)	23 (45.1%)	225 (29.9%)	434 (69.7%)	68 (62.4%)	*p* = 0.001 L1-3, *P* < 0.0001 ALL
No	142 (79.8%)	28 (54.9%)	528 (70,1%)	189 (30.3%)	41 (37.6%)	
**Stool consistency (** ***n*** **=)**	174 (96%)	48 (92%)	741 (95%)	624 (96%)	110 (99%)	
Shaped	96 (55.2%)	18 (37.5%)	271 (36.6%)	174 (27.9%)	30 (27.3%)	*p* = 0.001 L1-3, *P* < 0.0001 ALL
Mushy	53 (30.4%)	21 (43.7%)	298 (40.2%)	221 (35.4%)	36 (32.7%)	
Liquid	25 (14.4%)	9 (18.8%)	172 (23.2%)	229 (36.7%)	44 (40.0%)	
**Bowel movement per day (** ***n*** **=)**	131 (72%)	41 (79%)	636 (81%)	506 (77%)	90 (81%)	
0–2	88 (67.2%)	27 (65.9%)	326 (51.3%)	176 (34.8%)	33 (36.7%)	*p* = 0.001 L1-3, *P* < 0.0001 ALL
3–5	31 (23.6%)	11 (26.8%)	208 (32.7%)	182 (36.0%)	35 (38.9%)	
6–8	9 (6.9%)	2 (4.9%)	70 (11.0%)	80 (15.8%)	11 (12.2%)	
>8	3 (2.3%)	1 (2.4%)	32 (5.0%)	68 (13.4%)	11 (12.2%)	
**Bowel movement at night (** ***n*** **=)**	138 (76%)	36 (69%)	638 (82%)	507 (78%)	91 (82%)	
Yes	10 (7.2%)	8 (22.2%)	114 (17.9%)	138 (27.2%)	18 (19.8%)	*p* = 0.005 L1-3, *P* < 0.0001 ALL
No	128 (92.8%)	28 (77.8%)	524 (82.1%)	369 (72.8%)	73 (80.2%)	
**Anal findings (** ***n*** **=)**	158 (87%)	46 (88%)	703 (90%)	573 (88%)	100 (90%)	
None	136 (86.1%)	42 (91.3%)	549 (78.1%)	557 (97.2%)	97 (97.0%)	*p* = 0.011 L1-3, *P* < 0.0001 ALL
Fissures	16 (10.1%)	1 (2.2%)	93 (13.2%)	13 (2.3%)	1 (1.0%)	
Fistula/abscess	6 (3.8%)	3 (6.5%)	53 (7.6%)	2 (0.3%)	2 (2.0%)	
Inflammatory marisques	0	0	8 (1.1%)	1 (0.2%)	0	

*SD, standard deviation; n, number*.

Laboratory investigations at diagnosis showed no significant differences for albumin, hemoglobin, thrombocytes, lipase, and alanine aminotransferase (ALT) at diagnosis in patients with L2 compared with L1, L3, and UC (Supplementary Figure). The C-reactive protein (CrP) levels were significantly higher in patients with L2 at diagnosis; 6 and 12 months compared to UC (*p* = 0.009, *p* < 0.0001, *p* = 0.032, respectively), but not compared to IBD-U, L1 or L3 CD. The gamma-glutamyltransferase (GGT) values were significantly higher in L2 compared to L1 (*p* = 0.04), but not to L3, UC and IBD-U.

The weight standard deviation scores (SDSs), height SDS and BMI SDS at diagnosis was below the age- and gender-related median for all groups (Supplementary Figure). Weight SDS at diagnosis was significantly higher in patients with UC and IBD-U compared to L1 (*p* < 0.0001, *p* = 0.015), L2 (*p* < 0.001, *p* = 0.05) and L3 CD (*p* < 0.0001, *p* = 0.022), whereas there were no significant differences between the CD subgroups. The height SDS at diagnosis was significantly higher in the patients with UC than in the patients with L1 (*p* < 0.01) and L3 (*p* < 0.001) CD, but was not different from the patients with L2. In addition, BMI SDS at diagnosis is significantly higher in patients with UC than in those with L1 (*p* < 0.0001), L2 (*p* < 0.001), and L3 (*p* < 0.0001) CD patients.

### Extraintestinal Manifestations (EIM)

**Figure 1:**
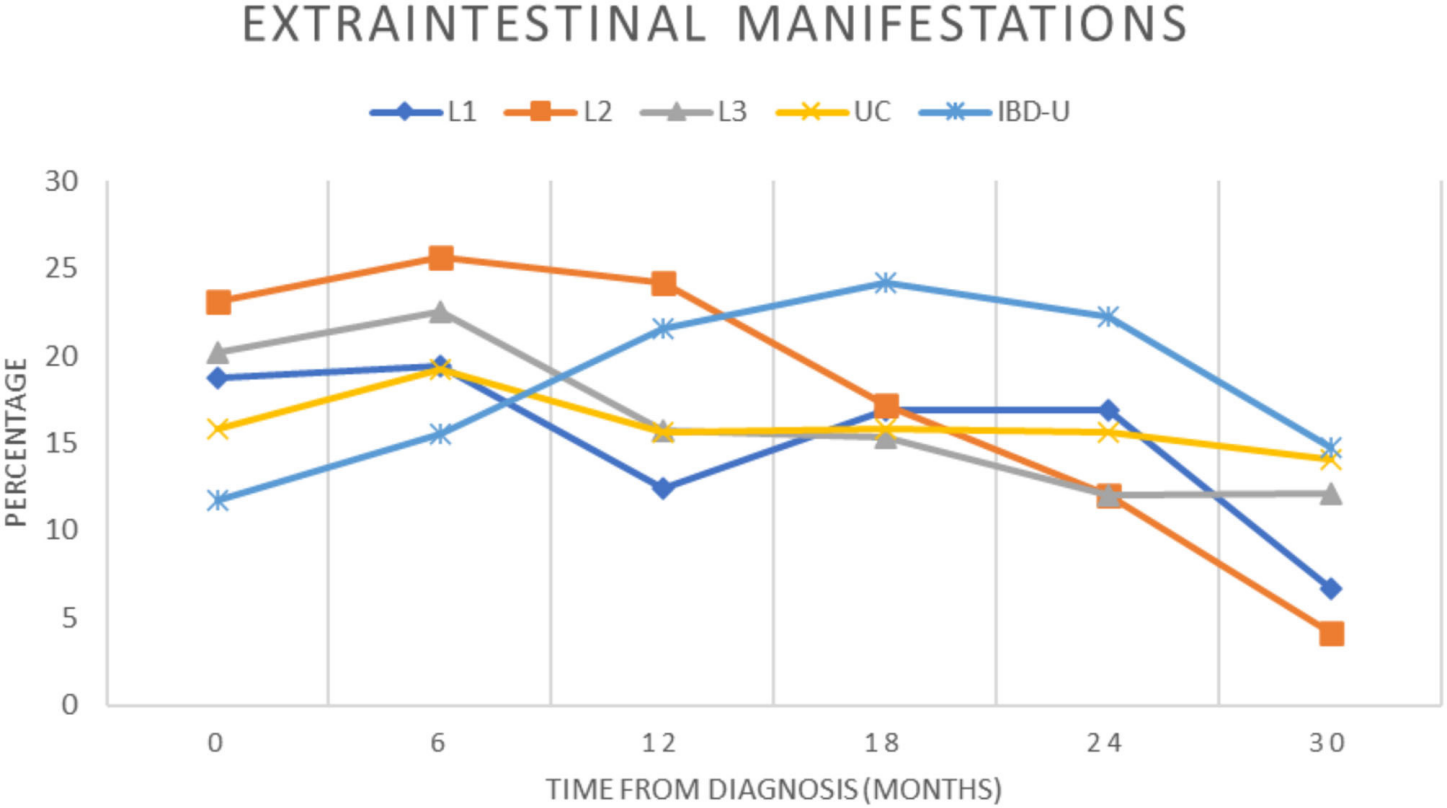
Presence of extraintestinal manifestations during the course of disease. The proportion of patients in the cohort with extraintestinal manifestations were recorded over a 2.5-year period for each subgroup and indicated from diagnosis (0) and for every 6 months (±3 months) of follow-up (L1, blue diamond; L2, orange square; L3, gray triangle; UC, yellow cross; IBD-U, light blue star).

Extraintestinal manifestations (EIM) in patients with PIBD at diagnosis were more frequent in L2 (23.1%) vs. L1 (18.7%, *p* = 0.48), L3 (20.2%, *p* = 0.6), CU (15.8%, *p* = 0.17), and IBD-U (11.7%, *p* = 0.061), but did not reach the significance. Joint involvement (peripheral and axial) was predominating in half of the patients with L2, compared with 44% for L1, 47% for L3, 25% for UC and 46% for IBD-U, followed by hepatobiliary (33%) and skin manifestations (25%) (Table 2). The percentage of EIM was declining after the first year of disease in patients with L2 while patients with IBD-U showed an increase of EIM (Figure 1). Primary sclerosing cholangitis (PSC) was not diagnosed in any of the patients with L2 CD, but in 28 CU, 4 L3 CD, 2 L1 CD, and 1 IBD-U. In contrast, EIM with liver involvement was found significantly increased in patients with L2 compared to L1 or L3. In addition, pancreatitis was found significantly more often in patients with L2 than in L1, L3, UC, and IBD-U. Furthermore, EIM of the skin was significantly more frequent in L2 compared to UC and IBD-U.

**Table 2:** Extraintestinal manifestations with diagnosis.

	**L1**	**L2**	**L3**	**UC**	**IBD-U**
	**(*N* = 182)**	**(*N* = 52)**	**(*N* = 782)**	**(*N* = 653)**	**(*N* = 111)**
**Extraintestinal involvement**	**34 (18.7%)**	**12^#a,b,c,d^ (23.1%)**	**158 (20.2%)**	**103 (5.81%)**	**13 (11.7%)**
Joints (peripheral arthritis or axial arthropathy)	15 (44.1%)	6 (50%)	75 (47.4%)	26 (25.2%)	6 (46.2%)
Liver/biliary tract/pancreas	3 (8.8%)	4 (33.3%)	17 (10.8%)	55 (53.4%)	4 (30.8%)
Skin (e.g., erythema nodosum)	5 (14.7%)	3 (25.0%)	6 (3.8%)	9 (8.7%)	1 (7.7%)
Other	11 (32.4%)	2 (16.7%)	60 (38.0%)	13 (12.6%)	2 (15.4%)

# *Not significant p > 0.05 Student's t-test*.

a *L2 vs. L1*.

b *L2 vs. L3*.

c *L2 vs. UC*.

d *L2 vs. IBD-U*.

### Induction Therapy

In total, 1.678 of the patients (94.3%) who received induction therapy were evaluated and the respective treatments are listed in Table 3. Significantly fewer patients with L2 CD (10.2%) received exclusive enteral nutrition (EEN) therapy as induction treatment compared to patients with L1 (34.3%, *p* = 0.001, RR3.4, 95%CI 1.53–7.92) and L3 (33.3%, *p* = 0.0008, RR3.3, 95%CI 1.52–7.55). Mesalazine or sulfasalazine treatment was mainly used for induction therapy in UC (76%) and IBD-U (75.5%) and significantly less common in patients with L2 (12.2%, *p* < 0.0001). Systemic cortisone treatment was used to induce remission in 40.8% of patients with L2 and was not significantly different from L1, L3, UC, and IBD-U. Immunomodulatory therapy was started within the first 3 months in 22.4% of patients with L2, mainly with azathioprine (81.8%). There was no statistically significant difference in between IBD or CD subgroups in this respect. Biologicals as an induction therapy were used only occasionally in all subgroups (Table 3).

**Table 3:** Induction therapy after diagnosis according to the disease groups.

	**L1**	**L2**	**L3**	**UC**	**IBD-U**
	**(*N* = 182)**	**(*N* = 52)**	**(*N* = 782)**	**(*N* = 653)**	**(*N* = 111)**
**Receive induction therapy**	**166 (91.2%)**	**49 (94.2%)**	**741 (94.8%)**	**620 (94.9%)**	**102 (91.9%)**
Antibiotics	15 (9.0%)	7 (14.3%)	90 (12.1%)	43 (6.9%)	14 (13.7%)
Mesalazine/sulfasalazine	73 (44.0%)	6 (12.2%)^*a,b,c,d^	410 (55.3%)	471 (76.0%)	77 (75.5%)
Exclusive enteral nutrition therapy (EEN)	57 (34.3%)	5 (10.2%)^*a,b^	247 (33.3%)	15 (2.4%)	10 (9.8%)
**Corticosteroids**					
Systemic	61 (36.7%)	20 (40.8%)	271 (36.6%)	251 (40.5%)	28 (27.5%)
Rectal	1 (0.6%)	3 (6.1%)	16 (2.2%)	18 (2.9%)	3 (2.9%)
**Immunomodulators**	18 (10.8%)	11 (22.4%)	242 (32.7%)	135 (21.8%)	11 (10.8%)
Tacrolimus	0	0	0	0	0
Methotrexate	0	1 (9.1%)	6 (2.5%)	2 (1.5%)	1 (9.1%)
Azathioprine	16 (88.9%)	9 (81.8%)	235 (97.1%)	123 (91.1%)	10 (90.9%)
Cyclosporin A	2 (11.1%)	1 (9.1%)	1 (0.4%)	10 (7.4%)	0
**Biologicals**	3 (1.8%)	1 (2.0%)	36 (4.9%)	7 (1.1%)	2 (2.0%)
Adalimumab	0	0	1 (2.8%)	0	0
Infliximab	3 (100%)	1 (100%)	29 (80.5%)	5 (71.4%)	2 (100%)
Other	0	0	6 (16.7%)	2 (28.6%)	0

* *p < 0.05 Student's t-test*.

a *L2 vs. L1*.

b *L2 vs. L3*.

c *L2 vs. UC*.

d *L2 vs. IBD-U*.

### Therapy in the Further Course of the Disease

After induction therapy, 42.3% of patients with L2 received immunomodulators and 21% biologics during follow-up (L1 56.5/10.5%; L3 59/21%; CU 43.5/11.9%; IBD-U 26.1/12.6%) (Table 4). The relative risk to receive immunosuppressants (IS) was lower in L2 and differed significantly from L1 (RR 0.71, 95% CI 0.49–0.96; χ^2^ *p* = 0.025) and L3 subgroup (RR 0.68, 95% CI 0.48–0.90; χ^2^ *p* = 0.005). There was no significant difference in terms of IS compared to UC and IBD-U (*p* > 0.05). The relative risk to be treated with biological agents was higher in patients with L2 compared to L1 (RR 2.02, 95% CI 1.018–3.895; χ^2^ *p* = 0.044). Almost three quarters of patients treated with an anti-TNF-alpha agent also present with EIM. Less patients with L2 (17.3%) received systemic steroids similar to IBD-U (IBD-U 15.2%), but this was not statistically significant compared to the other subgroups (L1 27.9%, L3 25.2%; UC 24%). Almost two-thirds of patients with L2 CD received mesalazine during the course, but this remained less frequent than in L3 CD, UC and IBD-U.

**Table 4:** Treatments of the respective disease groups in the course after induction therapy.

	**L1**	**L2**	**L3**	**UC**	**IBD-U**
	**(*N* = 182)**	**(*N* = 52)**	**(*N* = 782)**	**(*N* = 653)**	**(*N* = 111)**
**Any kind of therapy**	**172 (94.5%)**	**52 (100%)**	**743 (95.0%)**	**616 (94.3%)**	**99 (89.2%)**
Exclusive enteral nutrition (EEN)	16 (9.3%)	5 (9.6%)	64 (8.6%)	6 (1.0%)	4 (4.0%)
Mesalazine	95 (55.2%)	32 (61.5%)	521 (70.1%)	514 (83.4%)	75 (75.8%)
Sulfasalazopyridine	16 (9.3%)	11 (21.2%)	131 (17.6%)	145 (23.5%)	13 (13.1%)
**Immunosuppressants**	103 (59.9%)	22 (42.3%)^*a,b^	465 (62.6%)	284 (46.1%)	29 (29.3%)
Tacrolimus	0	0	0	1 (0.4%)	0
Methotrexate	11 (10.7%)	4 (18.2%)	63 (13.5%)	15 (5.3%)	2 (6.9%)
Azathioprine	90 (87.4%)	17 (77.3%)	392 (84.3%)	250 (88.0%)	27 (93.1%)
6- Mercaptopurine	2 (1.9%)	1 (4.5%)	8 (1.7%)	4 (1.4%)	0
Cyclosporin A	0	0	1 (0.2%)	14 (4.9%)	0
Mycophenolate mofetil	0	0	1 (0.2%)	0	0
**Biologicals**	18 (10.5%)	11 (21.2%)^*a^	165 (22.2%)	78 (12.7%)	14 (14.1%)
Adalimumab	5 (27.8%)	3 (27.2%)	33 (20.0%)	4 (5.1%)	4 (28.6%)
Infliximab	12 (66.7%)	7 (63.6%)	121 (73.3%)	55 (70.5%)	9 (64.3%)
Vedolizumab	0	0	2 (1.2%)	8 (10.3%)	0
Other	1 (5.6%)	1 (9.1%)	9 (5.5%)	11 (14.1)	1 (7.1%)
**Corticoids**					
Syst.	48 (28.0%)	9 (17.3%)	187 (25.2%)	148 (24.0%)	15 (15.2%)
Rect.	0	1 (1.9%)	7 (0.9%)	12 (1.9%)	2 (2.0%)

* *p < 0.05 Student's t-test*.

a *L2 vs. L1*.

b *L2 vs. L3*.

### Surgery

The number of patients requiring surgery did not differ significantly within the CD subgroups (L1 = 14.8%; L2 = 15.4%; L3 = 17.4%) and was significantly lower in UC and IBD-U (3.2%, *p* < 0.0001; 2.7%, *p* = 0.0019). The first surgery was performed after diagnosis at a median of 20 (SD ± 17) months in patients with L2, 20.5 (SD ± 26) months in UC; 13 (SD ± 33) months in L1; 6 (SD ± 39) months in L3; and 3 (SD ± 0) months in IBD-U. The relative risk for surgery in patients with L2 compared to UC und IBD-U is higher (RR4.21, 95% CI 2.48–7.1; RR 5.0, 95% CI 1.68–15.29). Perianal fistula surgery was significantly more common in L2 (44%; 8/18) than in L1 (4.8%; 3/63, *p* = 0.0002) or L3 (21.7%; 78/359, *p* = 0.0395), no other fistula surgeries were performed. All eight patients with L2 who received surgery were included in the registry before 2016. Three of the eight patients also received anti-TNF alpha treatment. The relative risk for surgery due to perianal fistula compared to L1 is 9.33 (95% CI 2.94–29.73) and to L3 is 2.05 (95% CI 1.1–3.24). In contrast, enterocutaneous fistula surgery was performed in patients with L1 CD in 7.9% (5/63) and non-perianal fistula surgery including enterocutaneous, enteroenteric and blind-ending fistula was performed in L3 CD in 8.6% (31/359). At the same time, the frequency of surgery for perianal abscesses was also higher in patients with L2 (55.6%) than in L1 (12.7%, *p* = 0.0004) or L3 (38.4%, *p* > 0.05).

### Assessment of Disease Activity at Time of Diagnosis and During Follow-Up

**Figure 2:**
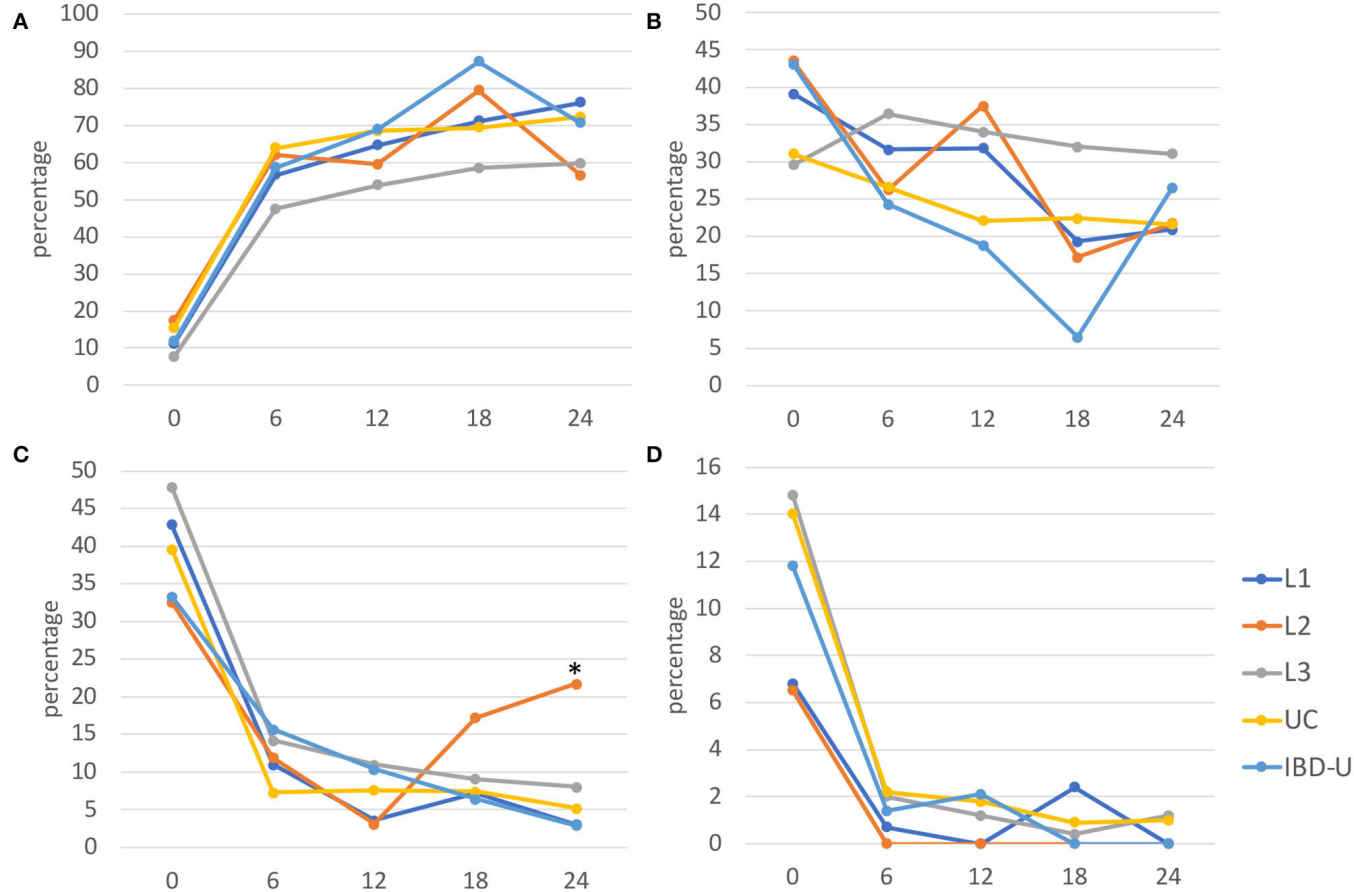
Disease activity from diagnosis to 24 months after. Disease activity was evaluated at diagnosis (0), and for every 6 months (±3 months) of follow-up for each subgroup using the physician global assessment (PGA) and presented as percentage for **(A)** remission, **(B)** mild, **(C)** moderate, and **(D)** severe activities (L1, blue; L2, orange; L3, gray; UC, yellow; IBD-U, light blue). Significant differences (*p < 0.05) were found for moderate disease activity in L2 after 24 months compared with the other subgroups, respectively.

Physician global assessment (PGA) at time of diagnosis showed a trend to a less moderate-to-severe phenotype in patients with L2 at diagnosis compared to UC (*p* = 0.058) and L3 (*p* = 0.0015*)* (Figure 2). Comparable remission rates were achieved in all subtypes after 6, 12, 18, and 24 months, with no significant differences. The relative risk of moderate-to-severe disease activity 24 months after diagnosis is higher for patients with L2 than for L1 (RR 7.5; 95% CI 1.77–31.76), L3 (RR 2.37; 95% CI 1.0–4.91), UC (RR 3.51; 95%CI 1.41–7.92), and IBD-U (RR7.39; 95% CI 1.24–46.13).

**Figure 3:**
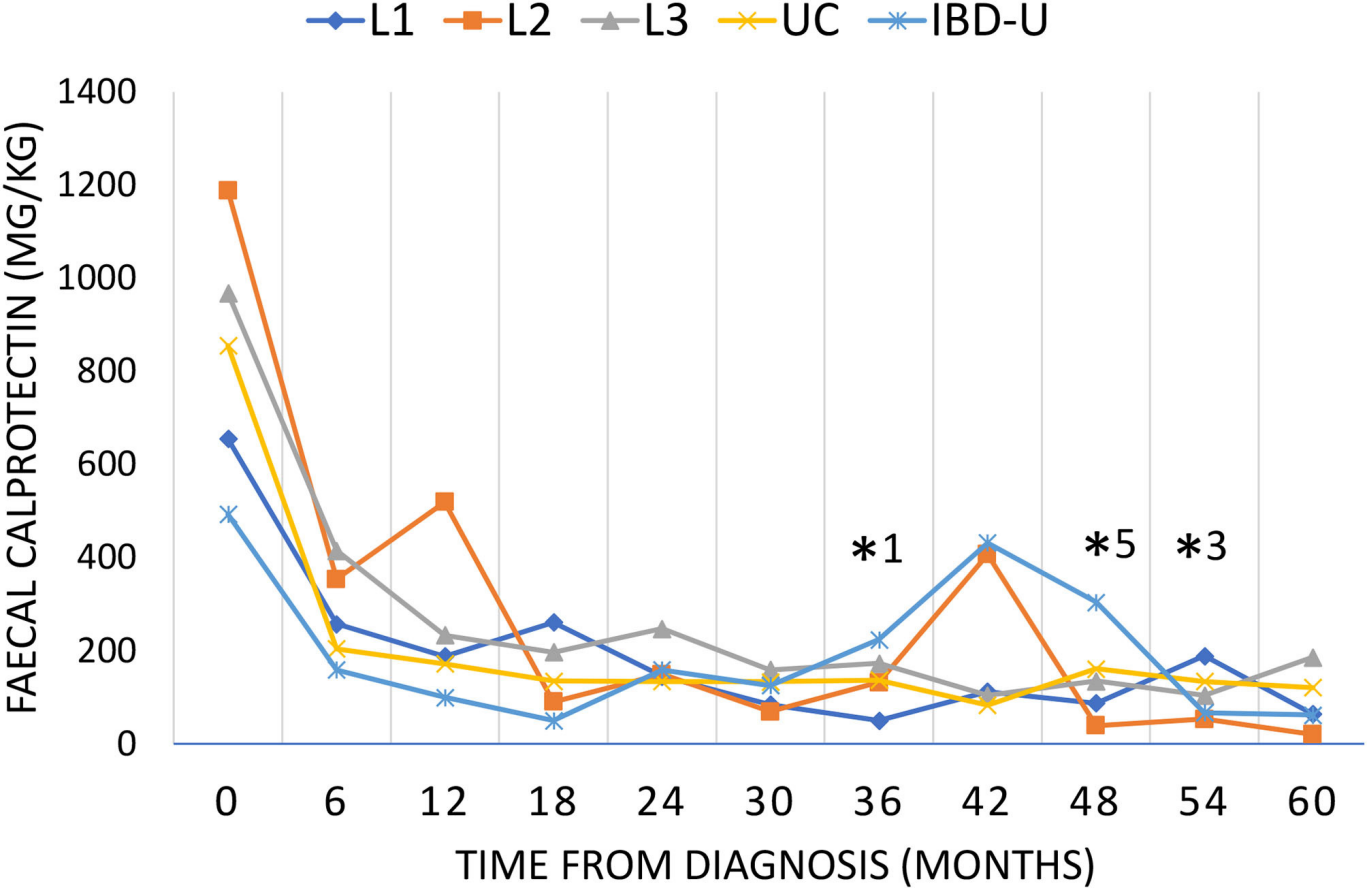
Fecal calprotectin during the course of disease. Disease activity was evaluated by fecal calprotectin every 6 months for up to 60 months as long as the patients have been followed up. Fecal calprotectin is presented as median for each subgroup (L1, blue diamond; L2, orange square; L3, gray triangle; UC, yellow cross; IBD-U light blue star). Significant differences (*p* < 0.05) were found for L2 compared to L1*^1^, IBD-U*^5^ and L3*^3^.

The median fecal calprotectin value was initially higher in patients with L2 (ranging from 137–2,100 mg/kg) and fell to comparatively lower values than in other subgroups after the first year after diagnosis (Figure 3). A second increase in fecal calprotectin was observed at 36 months after diagnosis in L2 and IBD-U, but not in L1, L3, and UC. The fecal calprotectin then again reaches low values as in the other groups.

## Discussion

We present comparative data from a large real-world dataset including patients with L1, L2, L3, UC, and IBD-U from 112 centers across Germany and Austria from 2004 to 2020. Our study complements data on presentation at diagnosis and treatment, disease activity, extraintestinal manifestations, and surgery of isolated pediatric colonic Crohn's disease.

In terms of clinical features our dataset puts patients with L2 CD in between L1 and UC, as suggested by a previous study of pediatric patients with L1, L2, and UC (21). Patients with L2 CD show bloody stools more frequently than L1 and L3, but less frequently than UC and IBD-U, and share other clinical and laboratory characteristics with these entities, e.g., a higher CrP level in L2 as in L1 and L3 CD compared to UC and IBD-U.

It is important to more clearly delineate these disease entities, as IBD-U phenotyping usually results in the exclusion of patients from studies, suggesting greater accuracy in disease phenotyping that does not exist in reality. This was also recognized by the Pediatric IBD Porto group, who revisited the diagnostic criteria for IBD subtypes in 2017, with special attention to the colonic phenotypes (22). They categorized class-1 (incompatible with UC), class-2 (suggestive of CD) and class-3 features (suggestive but have been found in UC in 5–10%) (22). An important clinical criterion for distinguishing L2 from UC is skip lesions or other segmental abnormalities to classic UC localization, which is also the case in our cohort, as a large percentage of patients with L2 have discontinuous colonic involvement.

Using leveraged machine learning, a Canadian group was able to define seven features which discriminate between UC and colonic CD in a small cohort (23). The use of data from large registries can contribute to the research, validation and further improvement of distinguishing criteria of UC, IBD-U, and L2-CD.

Isolated Crohn's colitis dominates in early onset Crohn's disease with almost half of the cases (10). The proportion of L2 declines with age in PIBD from 34% below 10–11% from 10 years and older (6). In contrast, isolated colonic Crohn's disease was found associated with older age at presentation in adults (19). Our evaluation confirms that some L2 cases occurred in early childhood compared to L1 CD and the children are slightly younger on average. Ileal involvement increases with age and is associated with CARD15/NOD2 polymorphisms suggesting that other genetic factors play a role in L2 (5, 8, 24). Indeed, the largest genotype–phenotype study of IBD in adults showed that ileal and colonic CD are at least as genetically distinct from each other as they are from UC (12).

Colonic involvement is an independent risk factor for multiple EIM in adults with IBD (25). Assa et al. analyzed long-term outcome of 301 pediatric patients according to the Paris classification and found that colonic involvement is associated with an increased rate of extraintestinal manifestations (L2 33%, vs. L1 18% and L3 13%) (6). Our data also show that EIM developed frequently in L2. Particularly relevant in patients with L2 CD are EIM of joint, skin, hepatobiliary, and pancreas according to our study. This should be considered when choosing therapeutic strategies because of associated long-term implications and morbidity.

Concerning hepatobiliary involvement, pancolitis and atypical CU with rectal sparing dominate in 65% and in 30% of patients with CU with PSC, which is much more frequent than in CU without PSC (50/10%) (26). Despite the previously described common genetic susceptibility profile linking PSC more strongly to CU than to CD, only half of the PSC risk loci overlap with IBD risk loci and half with other autoimmune diseases. The known genetic risk factors do not account for more than 10% of PSC susceptibility, leaving much room for environmental factors to be associated with the colon disease (27). Already 2012 Boonstra et al. demonstrated the role of colonic involvement as distinct phenotypical risk factor for PSC also in CD (28). A causal relationship may exist for translocation of bacterial components through an inflamed and leaky colon into the portal circulation and associated liver inflammation through macrophages and gut-derived T cells (29). Interestingly, we have not recorded any PSC in L2 CD in our pediatric registry.

Extraintestinal manifestations were found in 18% of patients in the registry at the time of diagnosis. In the pediatric Swiss IBD cohort, EIM occurred in 16.7 % of patients with IBD, and these patients were significantly more likely to be treated with anti-TNF-alpha agents than those without EIM (9). In this study, response rates to anti-TNF-alpha treatment in peripheral arthritis were highest at 61%. We found patients with L2 had joint involvement in up to half of the cases with EIM. This explains the comparatively high rate of anti-TNF-alpha therapy in this group.

Low fecal calprotectin levels indicate mucosal healing, which is associated with sustained clinical remission, lower hospitalization rates and fewer surgical procedures, in both CD and UC (30–33). Despite higher fecal calprotectin levels at diagnosis, disease activity by PGA was graded less severe in L2 CD than in CU and IBD-U. In contrast, in the study by Berger at al. more patients with L2 CD had severe inflammatory activity (recorded with the PCDAI) at the time of diagnosis (21). In the course, the median calprotectin levels normalized in all groups and comparatively good remission rates ranging from 54 to 69% were observed. Better remission rates of ~80% were achieved in the study by Berger et al., although it is important to bear in mind that our collective also includes many patients who were diagnosed many years ago and did not have the same treatment options (e.g., biologics) at that time (21).

We found that significantly less patients with L2 CD received EEN as an induction regiment, which has also been previously described in another cohort (21). This treatment decision could be due to the fact that patients with L2 responded less well to EEN (only 50% response in CD L2 compared to 82.1 and 91.7% in ileocolonic or ileal CD, respectively), but in a small cohort (L2 *n* = 14, L3 *n* =3 9, L1 *n* = 12) (17, 18). Other studies found conflicting results with similar efficacy for EEN independent of localization (21, 34–36). In our study, we found a relevant association of L2 CD with risk of EIM and risk for surgery for perianal disease. Extraintestinal manifestations and perianal disease have been shown to respond poorly to EEN, which partially explains the lower EEN use in our cohort. According to the recent recommendations, all patients with luminal CD should receive EEN regardless of localization pattern (35). Feedback on this is provided to the participating centers in the registry to favor treatment with EEN in patients with L2 without EIM.

As in the Berger et al. study, we found a high proportion of patients receiving immunomodulators for maintenance therapy and a significant proportion of patients receiving biologicals (21). However, the probability to receive immunosuppressive therapy is lower in L2 compared to L1 and L3 phenotype in our study. As previously mentioned, the frequent use of anti-TNF-alpha agents in our study can be explained by the frequency of EIM with joint involvement and perianal fistulas in patients with L2 (37, 38). Mesalazine was also administered significantly less frequently in our cohort compared to UC and IBD-U, as has also been reported by others (21). The lack of response of L2 toward mesalazine, which targets the epithelium surface, again indicates differences in the pathophysiology of Crohn's colitis and UC (19).

The cumulative risk of an adult having surgery within 10 years of diagnosis of isolated Crohn's disease is 22–33%. This is significantly lower than the 75–90% risk for ileal Crohn's disease (19). Partial or segmental colon resections or colectomy for pancolitis have been described as successful (19). In ulcerative colitis, it is not the extent but the severity at diagnosis that is associated with colectomy (6). In children with L2 at diagnosis the risk for receiving an intestinal resection is significantly reduced compared to L1 and L3 phenotypes (6). While patients with L2 in our cohort did not receive intestinal resections during the observation period, the risk of surgery due to perianal fistulae and abscesses was significantly increased. In fistulizing perianal disease anti-TNF-alpha treatment is recommended as induction and maintenance therapy (38), but historically surgical approaches were common. Our data is a long-term follow-up of some patients diagnosed more than 15 years ago. Therefore, the higher proportion of surgical treatment of perianal disease can be explained historically, as well as the higher proportion of patients who received anti-TNF-alpha treatment during follow-up.

The predictive property of phenotypic characterization at presentation according to the Paris classification of pediatric IBD has been shown (6). In addition, according to the genetic basis of IBD a classification into ileal CD, colonic CD, and ulcerative colitis has been suggested (12). Our data support the classification of CD phenotype L2 as a separate entity since it behaves like Crohn's disease on the one hand and like CU on the other. In summary, specific clinical pictures of L2 include: one finds elevated CrP levels (as with L1/3 CD) and high calprotectin (as with UC and L3) at diagnosis, and it also negatively affects height, weight and BMI at diagnosis, but more often leads to EIM and perianal surgery than L1 or L3. It also influences therapeutic management with less EEN, a slightly lower proportion of immunomodulators and a higher proportion of anti-TNF-alpha agents.

Our analysis has several limitations. The selection of patients from a registry containing data from many tertiary pediatric gastroenterological centers may bias the data toward more severe disease phenotypes and more aggressive initial therapeutic approach. In addition, it is important to consider that the proportion of L2 CD was 5.4% among the CD subgroups in our cohort, much lower than in other studies, where the proportion was 13–20%. Furthermore, the observation time differs considerably within the groups, which could lead to a bias in some evaluations. The registry does not contain genetic, video endoscopic or histological data, so the classification of disease by pediatric gastroenterologists is based on the relevant guidelines at the time of study, limiting the ability to retrospectively clarify classification conflicts. The strength of the long follow-up period in such a large, all-pediatric cohort is related to the bias of including historical data as treatment options and strategies (step up or top–down) change over time.

## Conclusions

The consideration of Crohn's colitis as a distinct disease group seems necessary. It is characterized by a phenotype between L3 CD, IBD-U, and UC. In particular, extraintestinal manifestations with joint involvement or perianal fistulas require more often anti-TNF-alpha therapy. The different IBD phenotypes and their pathogenesis most likely influence not only initial presentation but also their natural course, the response to therapeutics, and relevant long-term risks. A precise classification therefore is mandatory for all other studies in pediatric IBD. The phenotypical delineations will be more closely investigated in studies in the future, with the need of long-term follow-up and large real-world registry data.

## Data Availability Statement

The datasets presented in this article are not readily available because the CEDATA-GPGE registry as well its contained data are owned by the registered association “CEDATAGPGE”. Therefore, the propagation of data originating from the register can only be approved by the owner. Requests to access these datasets should be directed to https://www.gpge.eu/cedata-gpge.

## Ethics Statement

The studies involving human participants were reviewed and approved by Ethik-Kommission am Fachbereich Medizin der Justus-Liebig Universität Giessen, Gaffkystr. 11c, D-35385 Gießen. Written informed consent to participate in this study was provided by the participants' legal guardian/next of kin.

## CEDATA–GPGE Study Group Members

Tobias Schwerdt, Munich; Rainer Ganschow, Bonn; Stefan Trenkel, Potsdam; Burkhard Rodeck, Osnabrück; Stefan Wirth, Wuppertal; Marlen Zurek, Leipzig; Matthias Heiduk, Magdeburg; Michael Paulussen, Datteln; Gunter Flemming, Leipzig; Ekkehard Sturm, Tübingen; Axel Enninger, Stuttgart; Söhnke Dammann, Stuttgart; Henning Böhme, Quedlinburg; Michael Melter, Regensburg; Thomas Lang, Regensburg; Philip Bufler, Berlin; Thomas Lücke, Bochum; Markus Knuf, Worms; Norbert Wagner, Aachen; Thomas Kaiser, Münster; Ralf Pallacks, Memmingen; Andre Hörning, Erlangen; Jens Klinge, Fürth; Steffen Reinsch, Jena; Rüdiger Adam, Mannheim; Stefan Buderus, Bonn; Markus Richter, Augsburg; Antje Ballauf, Krefeld; Ilse Broekaert, Köln; Lars Heerdts, Cottbus; Carolin Blüml, Marburg; Sabine Peitzsch, Landshut; Andreas Krahl, Darmstadt; Simone Jedwilayties, Friedrichshafen; Maik Heine, Hoyerswerda; Marko Reitmann, Borna; Kai Nils Pargac, Meißen; Thomas Lang, Starnberg; Jutta Kringel, Bad Kreuznach; Anke Dick, Würzburg; Patrick Gerner, Freiburg; Michael Friedt, Düsseldorf; Enno Iven, Hamburg; Gunter Burmester, Hamburg; Anke Esser, Neuss; Olaf Raecke, Esslingen; Kerstin Ehrentraut, Altenburg; Esther Schmidt, Schwerin; Jan Däbritz, Rostock; Stefan Sgoll, Gründau; Ahlke Willenborg, Berlin; Sebastian Horn, Suhl; Ralph Melchior, Kassel; Rüdiger Kardoff, Duisburg; Martina Kohl-Sobania, Lübeck; Benedikt Pircher, Künzell; Christoph Ehrsam, Meiningen; Daniela Nolkemper, Hamburg; Adrian Lieb, Neu-Isenburg; Almuth Hauer, Graz; Markus Prenninger, Wels; Martin Laaß, Dresden; Dieter Furthner, Vöcklabruck.

## Author Contributions

CP is acting as the submission's guarantor and takes responsibility for the integrity of the work. CP, LW, and LE substantially contributed to conception and design of the study. LE, CP, and JL did analysis and interpretation of data and statistical analysis. CP and JL did writing-up of the first draft of the article. LE revising it critically for important intellectual content. All authors finally approved the version to be published.

## Funding

The CEDATA–GPGE has received research funding by Abbvie, Takeda, Dr. Falk. The CED–KQN project connected to this study was funded by the Gemeinsamer Bundesausschuss (Federal Joint Committee Germany, VSF17054).

## Conflict of Interest

The authors declare that the research was conducted in the absence of any commercial or financial relationships that could be construed as a potential conflict of interest.

## Publisher's Note

All claims expressed in this article are solely those of the authors and do not necessarily represent those of their affiliated organizations, or those of the publisher, the editors and the reviewers. Any product that may be evaluated in this article, or claim that may be made by its manufacturer, is not guaranteed or endorsed by the publisher.
