# Supplementary material for: Isolated Crohn's Colitis: Is Localization Crucial? Characteristics of Pediatric Patients From the CEDATA–GPGE Registry
Source: Front Pediatr. 2022 May 31;10:875938. doi: 10.3389/fped.2022.875938 (PMC9194809; doi:10.3389/fped.2022.875938)
Supplement: Supplementary file 1 [file Data_Sheet_1.pdf]

Auxological data at diagnosis

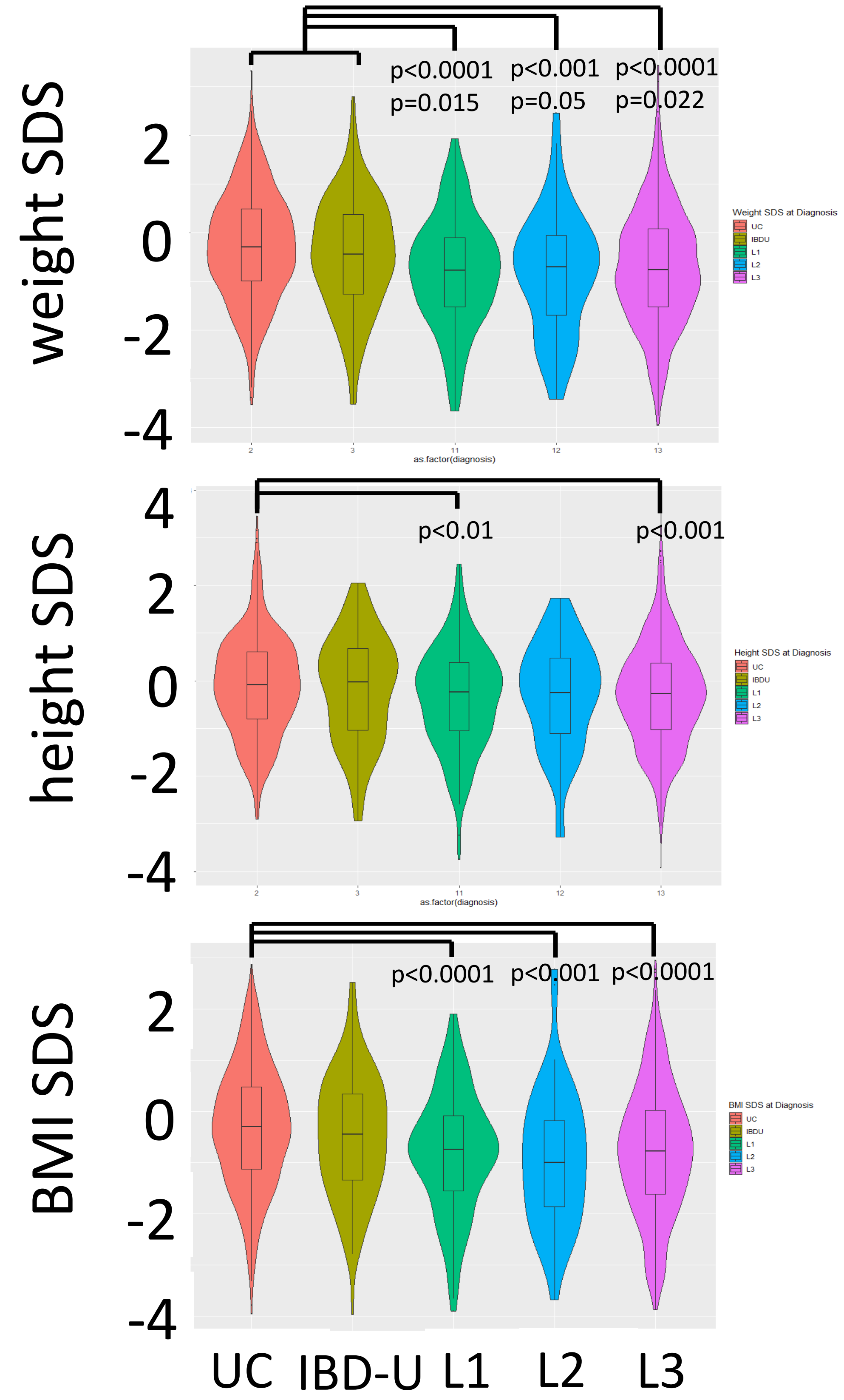

Weight standard deviation scores (SDS), height SDS and BMI SDS for each subgroup at diagnosis and after 6 and 12 months of follow up are indicated by a truncated violin blot as well as a box and whisker diagram. The box represents the first and third quartiles, the horizontal line inside indicates the median value. Significant differences between L2 and the other subgroups were indicated.

C-reactive Protein

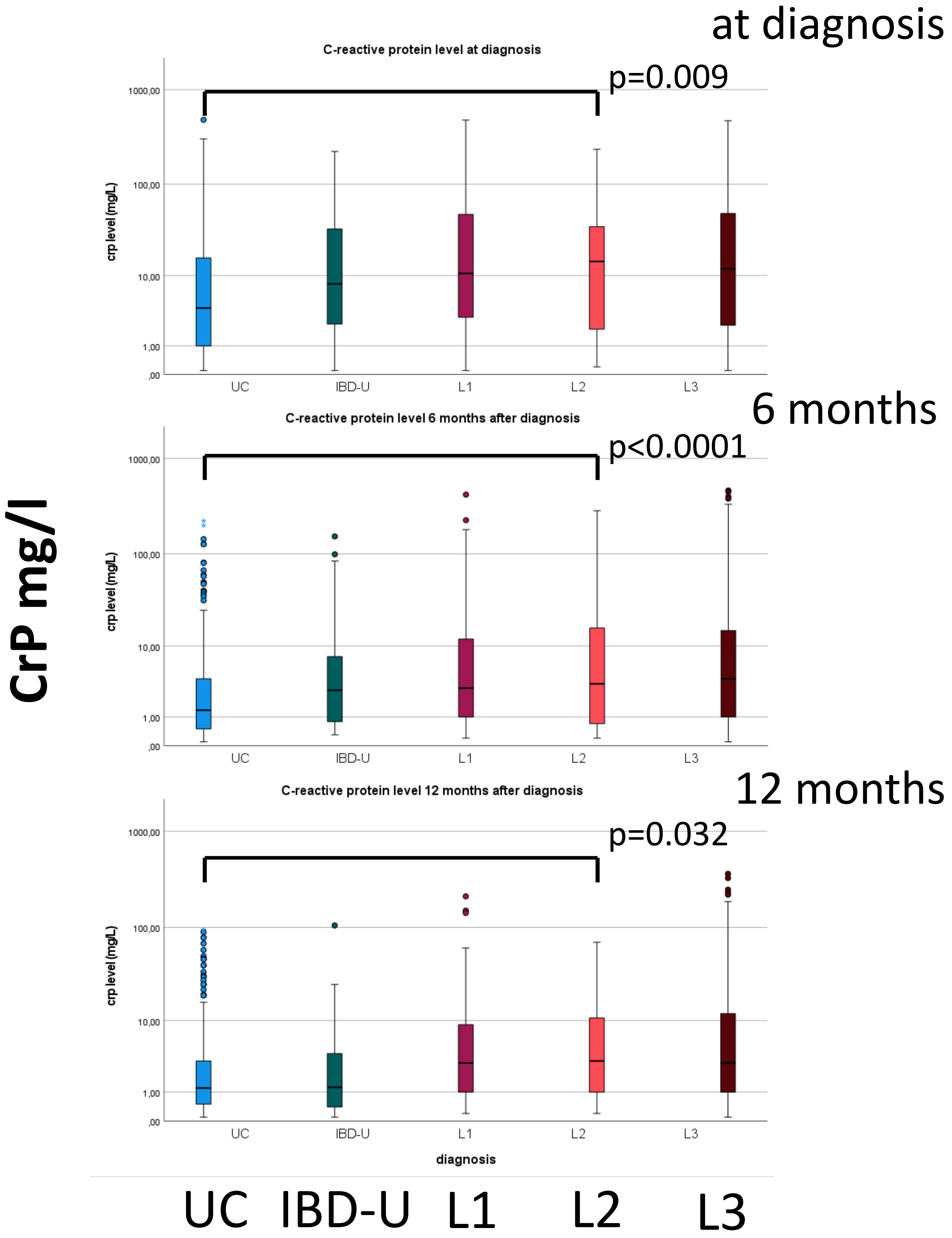

C reactive Protein (CrP) values in mg/l for each subgroup at diagnosis and after 6 and 12 months of follow up are indicated by a box and whisker diagram. The box represents the first and third quartiles, the horizontal line inside indicates the median value. Significant differences between L2 and the other subgroups were indicated.

Haemoglobin

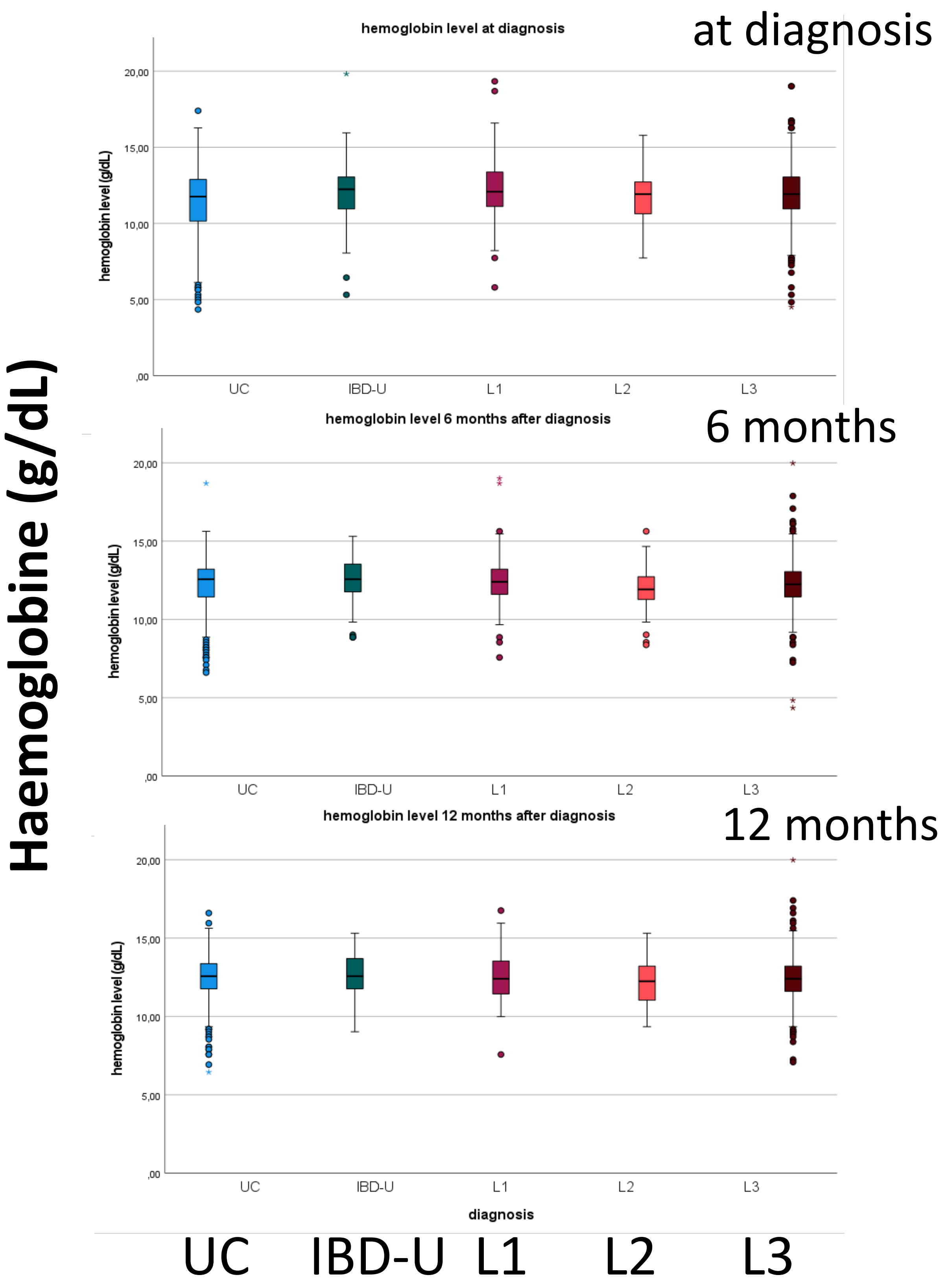

Haemoglobin values in g/dl for each subgroup at diagnosis and after 6 and 12 months of follow up are indicated by a box and whisker diagram. The box represents the first and third quartiles, the horizontal line inside indicates the median value. No significant differences ($p<0.05$) between L2 and the other subgroups were found.

Thrombocytes

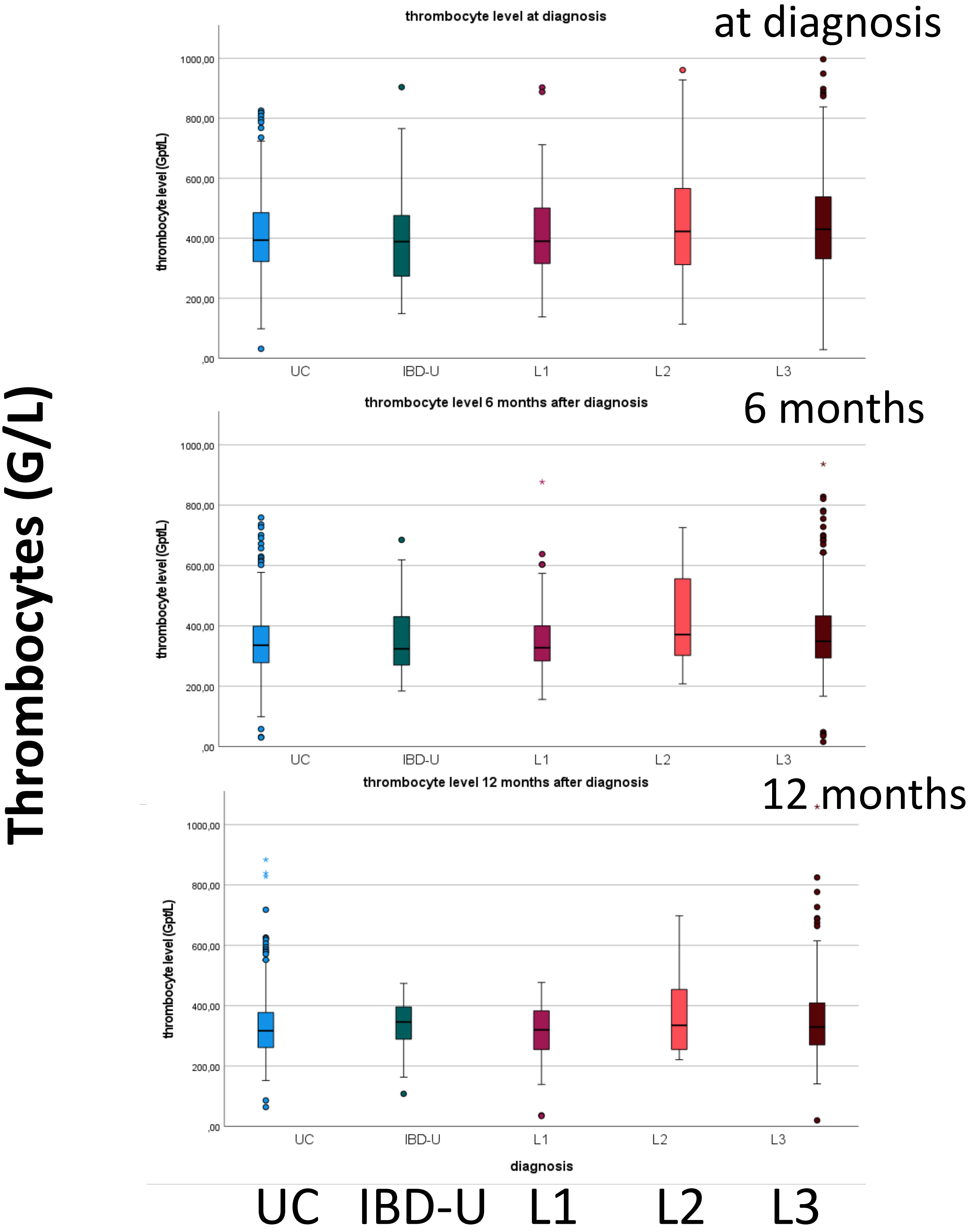

Thrombocyte counts in G/l for each subgroup at diagnosis and after 6 and 12 months of follow up are indicated by a box and whisker diagram. The box represents the first and third quartiles, the horizontal line inside indicates the median value. No significant differences ($p<0.05$) between L2 and the other subgroups were found.

Lipase

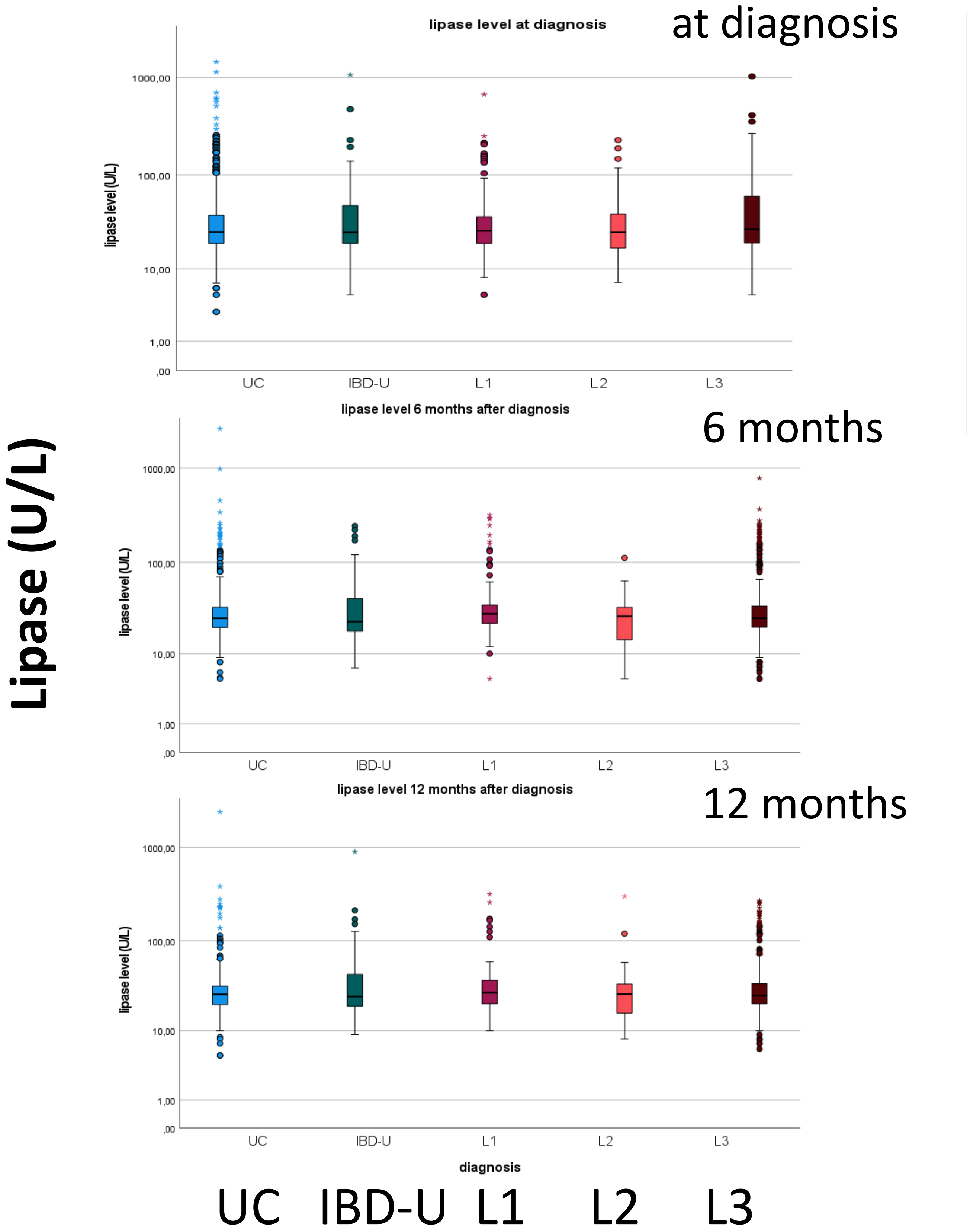

Lipase level in U/l for each subgroup at diagnosis and after 6 and 12 months of follow up are indicated by a box and whisker diagram. The box represents the first and third quartiles, the horizontal line inside indicates the median value. No significant differences ($p<0.05$) between L2 and the other subgroups were found.

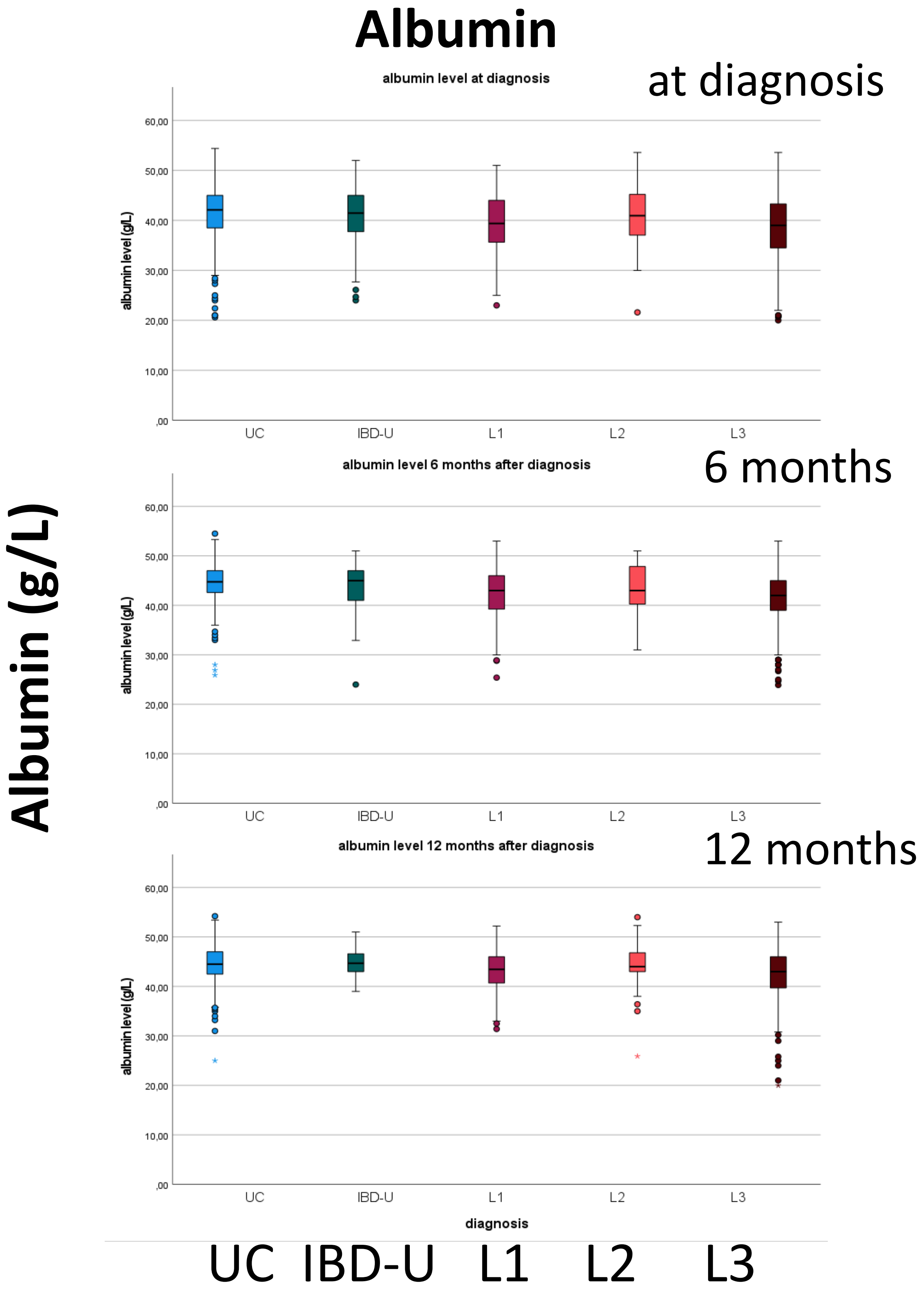

Albumin level in g/l for each subgroup at diagnosis and after 6 and 12 months of follow up are indicated by a box and whisker diagram. The box represents the first and third quartiles, the horizontal line inside indicates the median value. No significant differences ($p<0.05$) between L2 and the other subgroups were found.

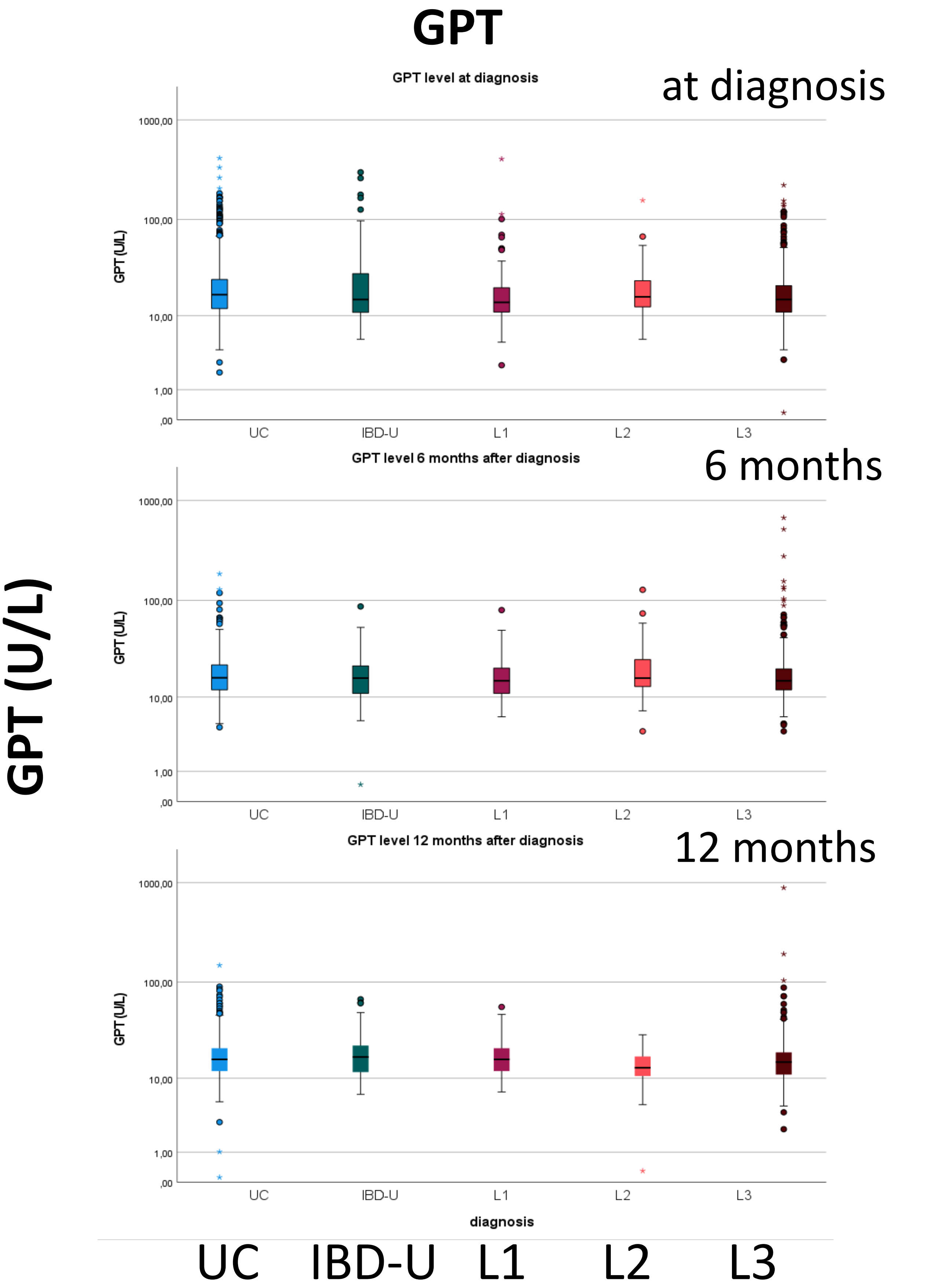

Glutamate-pyruvate transaminase (GPT) level in U/l for each subgroup at diagnosis and after 6 and 12 months of follow up are indicated by a box and whisker diagram. The box represents the first and third quartiles, the horizontal line inside indicates the median value. No significant differences ($p<0.05$) between L2 and the other subgroups were found.

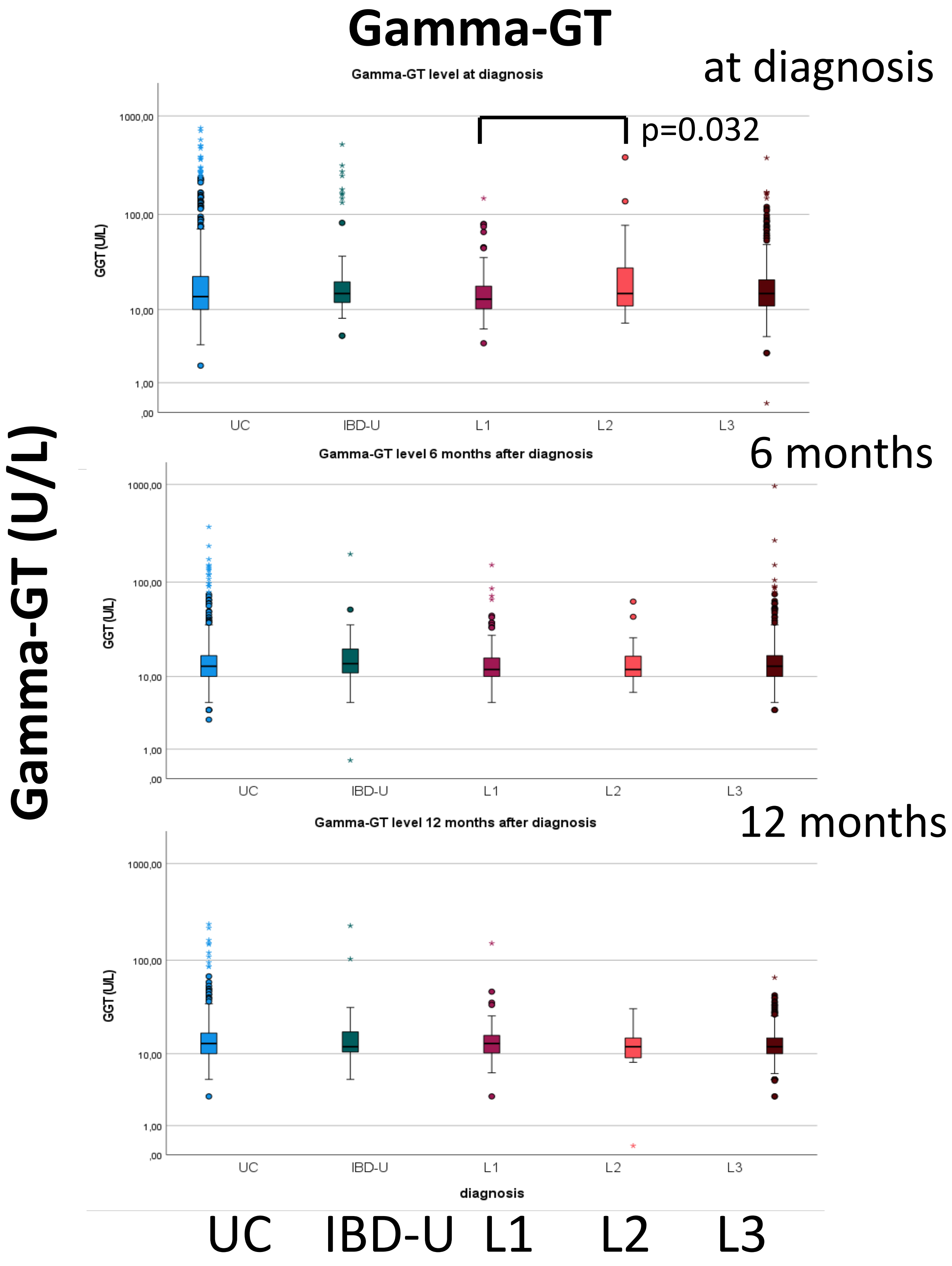

Gamma-glutamyl transferase (GGT) values in U/l for each subgroup at diagnosis and after 6 and 12 months of follow up are indicated by a box and whisker diagram. The box represents the first and third quartiles, the horizontal line inside indicates the median value. Significant differences between L2 and the other subgroups were indicated.
